# Can We Distinguish Emotions from Faces? Investigation of Implicit and Explicit Processes of Peak Facial Expressions

**DOI:** 10.3389/fpsyg.2016.01330

**Published:** 2016-08-31

**Authors:** Ruiqi Xiao, Xianchun Li, Lin Li, Yanmei Wang

**Affiliations:** School of Psychology and Cognitive Science, East China Normal UniversityShanghai, China

**Keywords:** intense emotion, unconscious perception, eye-tracking, ambiguous facial expression, affective priming

## Abstract

Most previous studies on facial expression recognition have focused on the moderate emotions; to date, few studies have been conducted to investigate the explicit and implicit processes of peak emotions. In the current study, we used transiently peak intense expression images of athletes at the winning or losing point in competition as materials, and investigated the diagnosability of peak facial expressions at both implicit and explicit levels. In Experiment 1, participants were instructed to evaluate isolated faces, isolated bodies, and the face-body compounds, and eye-tracking movement was recorded. The results revealed that the isolated body and face-body congruent images were better recognized than isolated face and face-body incongruent images, indicating that the emotional information conveyed by facial cues was ambiguous, and the body cues influenced facial emotion recognition. Furthermore, eye movement records showed that the participants displayed distinct gaze patterns for the congruent and incongruent compounds. In Experiment 2A, the subliminal affective priming task was used, with faces as primes and bodies as targets, to investigate the unconscious emotion perception of peak facial expressions. The results showed that winning face prime facilitated reaction to winning body target, whereas losing face prime inhibited reaction to winning body target, suggesting that peak facial expressions could be perceived at the implicit level. In general, the results indicate that peak facial expressions cannot be consciously recognized but can be perceived at the unconscious level. In Experiment 2B, revised subliminal affective priming task and a strict awareness test were used to examine the validity of unconscious perception of peak facial expressions found in Experiment 2A. Results of Experiment 2B showed that reaction time to both winning body targets and losing body targets was influenced by the invisibly peak facial expression primes, which indicated the unconscious perception of peak facial expressions.

## Introduction

Facial expression, which conveys affective and motivational states, serves as one of the most important nonverbal social cues in daily interpersonal communication. Thus, the ability to extract emotion information from facial expression is crucial for efficient social functioning and interpersonal relationships (Hinojosa et al., 2015). There are two important processes of facial expression recognition: explicit recognition and implicit perception. Implicit facial expression perception, occurring relatively quickly, can be made with limited information input and without consciousness. Conversely, explicit facial expression recognition requires comparison between the currently obtained features and related prior knowledge (Landis, 1924; Adolphs, 2002). Various evidence has been provided to support the notion that implicit and explicit processes are distinct and independent. For example, the adult neuroimaging literature suggests different underlying neural structures for these two processes: subcortical limbic activity for the implicit process and the response of the prefrontal cortex for the explicit process (Nakamura et al., 1990; Joynt, 1995; Winkielman et al., 1997; Adolphs, 2002; Lange et al., 2003). Moreover, other studies have demonstrated that the strength of activation of the amygdala differed between implicit perception and explicit recognition processes, although no consensus on how the activation changes was obtained (Studies revealing enhanced reaction of amygdala in implicit facial emotion perception, see: Williams et al., 2005; Habel et al., 2007, Studies revealing less response in implicit facial expression perception, see: Gorno-Tempini and Price, 2001; Gur et al., 2002).

Most studies of facial emotion recognition have focused on basic emotions of moderate intensity (Ekman and O'sullivan, 1988; Ekman, 1993; Young et al., 1997; Smith et al., 2005). Although, debate continues (Gendron et al., 2014), most studies using moderate intensity facial expressions have revealed that six basic emotions (happiness, sadness, disgust, fear, anger, and surprise) are universal, and people can automatically and accurately recognize or perceive them from face cues both explicitly and implicitly (Boucher and Carlson, 1980; Haidt and Keltner, 1999; Sauter et al., 2010; Ekman and Cordaro, 2011). However, apart from the well-recognized moderate emotions, there are many more facial expressions that are ambiguous in our daily life, such as peak emotion. Peak emotion is one kind of the unexploited emotions, which was defined by Aviezer et al. (2012b) as “*the apex of a highly intense emotional experience and focused on the immediate peak expressions in response to real-life situations, such as undergoing a nipple piercing, receiving an extravagant prize, winning a point in a professional sports match, and so forth.”* Some studies investigated intensity as an important factor to influence expression recognition, finding that recognition accuracy improved as expression intensity increased. However, we found that they did not actually take peak emotion into account, because the intensity of the stimuli they adopted was far below peak emotions, even for the most intensive stimuli (Orgeta and Phillips, 2007; Hoffmann et al., 2010; Leime et al., 2013; Rosenberg et al., 2015).

The current state of research on peak emotion is inadequate. According to the limited number of studies, peak emotions are unable to convey emotion information. To our knowledge, the work of Aviezer et al. (2012b) pioneered the investigation of recognition of peak expressions in real life situations. Their study employed expression images of athletes at the moment of winning or losing a point; the participants were asked to deduce the valence of isolated faces, isolated bodies, the congruent face-body compounds, and incongruent ones. The results showed that participants could judge the valence of isolated body images, though it was difficult for them to distinguish the isolated faces. Furthermore, the valence of incongruent face-body compounds was judged by body gestures, rather than facial expressions. They concluded that faces in intense situations were not capable of conveying emotion information. However, we need to be cautious about their conclusion considering the following perspectives.

First, we consider the communicatory function of facial expressions and discuss the diagnosability of peak facial expressions from the functional aspect. Facial expressions could convey specific information to observers, while simultaneously acting as reinforces to modulate further action (Blair, 2003). Although, peak facial expressions were distorted, facial expressions in peak emotional situations should still keep their communicatory characteristics. In many high-stake sporting competitions, athletes are required not to exhibit intensive expressions frequently since they are not necessarily functional in achieving goals (Friesen, 2015). For example, players rarely perform at their best when feeling sad. However, they nonetheless display some intense expressions. One possible reason for their conscious choice to express their feelings in an extreme way is to exaggerate their confidence, cheer themselves and their supporters up (for winners) or exhibit extreme anger to scare competitors (for losers).

Second, participants in Aviezer et al. study were asked to rate the valence of presented faces, which required consciously matching the obtained information and the existing experience. Thus, we cannot conclude that peak facial expressions are not capable of conveying emotion information, given that the implicit process was not tested. The results merely indicated that peak facial expressions cannot be recognized explicitly.

Third, the finding that the valence ratings of peak face-body compounds (congruent and incongruent) were mainly determined by body gestures is insufficient to support the notion that peak facial expressions are not diagnostic, because body cues also exert influence on emotion recognition in other situations where the faces conveyed strong and clear emotion information (Kret et al., 2013). For example, by using the facial expressions taken from Ekman and Friesen (1976) set, App et al. (2012) demonstrated that angry faces on fearful bodies were recognized as less angry than on angry bodies. The context influence, including bodily gestures, words, cultural context and voice (Barrett et al., 2011), on perception of facial expressions is thought to be automatic (Aviezer et al., 2011), outside consciousness (Aviezer et al., 2007, 2009, 2012a) and culturally unspecific (Ito et al., 2011).

Some expressions (such as anger and disgust) bear strong similarities in facial configuration, and the high degree of similarity could foster the influence of the body on expression recognition. Aviezer et al. (2008) highlighted the “similarity” between the facial configuration of different emotions, and suggested that the influence of the body on emotion recognition depends on the degree of similarity. In their study, they sought to find the influence of an angry body on emotional facial expressions. They found that when participants are presented with two images—one of an angry fist accompanied by a disgusted facial expression, the other of an angry fist accompanied by a fearful facial expression—they were more likely to choose the former image as anger, because of the high similarity of facial expressions between anger and disgust. In a similar vein, regarding peak facial expressions, the facial muscles tense to the greatest extent, making the faces of different peak emotions look alike so that the bodies can strongly influence emotion recognition.

Peak emotion is special, considering its anatomical structures and distorted appearance. Specific expression activates certain facial muscle combinations. For example, when smiling, the orbicularis oculi muscle and zygomaticus major muscles combine to raise the cheeks and the corners of mouth, while anger causes orbicularis oculi to lower, bringing the brows together, while orbicularis oris is caused to raise and tighten the upper eyelids. Theoretically, specific facial muscle combinations for each emotion and the way they work do not change as the intensity increases. However, for peak emotion, the facial muscles are extremely constrained, and the configuration distortion caused by high intensity makes the peak facial expressions hard to distinguish. Therefore, we query whether it is possible for observers to detect emotion information from the different facial muscle actions “hidden” under distorted facial configuration, in terms of facial muscle combination and the way they work.

The studies in this field are principally focused on explicit recognition, and there are few research studies investigating the implicit processes of peak emotion. Evidence for implicit emotion perception is mostly generated from studies using continuous flash suppression (CFS) techniques and the backward masking (BM) technique. These two paradigms are distinct regarding the strength of suppression and the underlying neural mechanisms activated. In CFS, the prime and noise are presented simultaneously to both eyes. The sequence of CFS is as follows: two fixation crosses appear on the screen, followed by the prime picture accompanied by the first random-noise pattern, followed by the same prime picture accompanied by the second random-noise pattern, followed by the targets. The noise images are usually presented to the dominant eye, and the facial expressions are presented to the other one (Adams et al., 2010). During binocular presentation, the dominant noise images obliterate the information of the suppressed image further up to the visual system, leaving the subcortical processing relatively unaffected (Tong and Engel, 2001). Subliminal affective priming task is an example of the backward masking paradigm. In this task, positive and negative primes are presented for a short time (17 or 30 ms), which could not be consciously detected, followed by a positive or negative target (Hermans et al., 2001). Participants were found to respond faster and more accurately to the targets when primed by congruent valence primes than when primed by incongruent valence primes. These two methods differ in the loci and degree of cortical activation. Almeida et al. (2013) reported that CFS were more sensitive to negative-valenced stimuli. Whereas, the major advance of the subliminal affective priming task is that its relatively “loose” masking procedure was proved to generalize activation across many cortical regions, demonstrating that it is sensitive to both positive and negative prime stimuli and eliminates the threat-specific effect of CFS.

Given previous findings, we have reasons to hypothesize that peak facial expressions could be implicitly perceived, even though the differences between peak facial expressions are too subtle to be explicitly recognized. Our study aimed to investigate the diagnosability of peak facial expressions at both conscious and unconscious levels. In Experiment 1, we investigated the explicit process of peak emotion. Participants rated the valence of isolated bodies, faces, congruent face-body compounds, and incongruent face-body compounds whilst their eye movement pattern was simultaneously recorded. Experiment 2A and 2B adopted the subliminal affective priming task to investigate the implicit process of peak facial expressions, with isolated bodies as the target and isolated faces as the prime. We hypothesized that the participants would fail to judge the peak facial expressions (Experiment 1), but would be able to implicitly perceive them, by showing the influence on reaction to other emotional body targets (Experiment 2A and 2B).

## Experiment 1

### Materials and methods

#### Participants

Thirty-two college students from East China Normal University (11 males and 21 females; *M* = 20.41 years, *SD* = 1.30, range: 19–24 years) participated in the experiment. All the participants were right-handed, had normal or corrected-to normal vision, and had no neurological or psychiatric history. They gave written informed consent and received small gifts for their participation. All the participants were included in the behavioral analyses. Nine of them (two males and seven females) were excluded from the eye movement data collecting procedure, due to the possible influence of spectacles. Of the remaining 23 participants, five participants in the face-body compound blocks were rejected due to technical problems. The study was approved by the Institutional Ethics Committee of East China Normal University.

#### Materials

**Figure 1:**
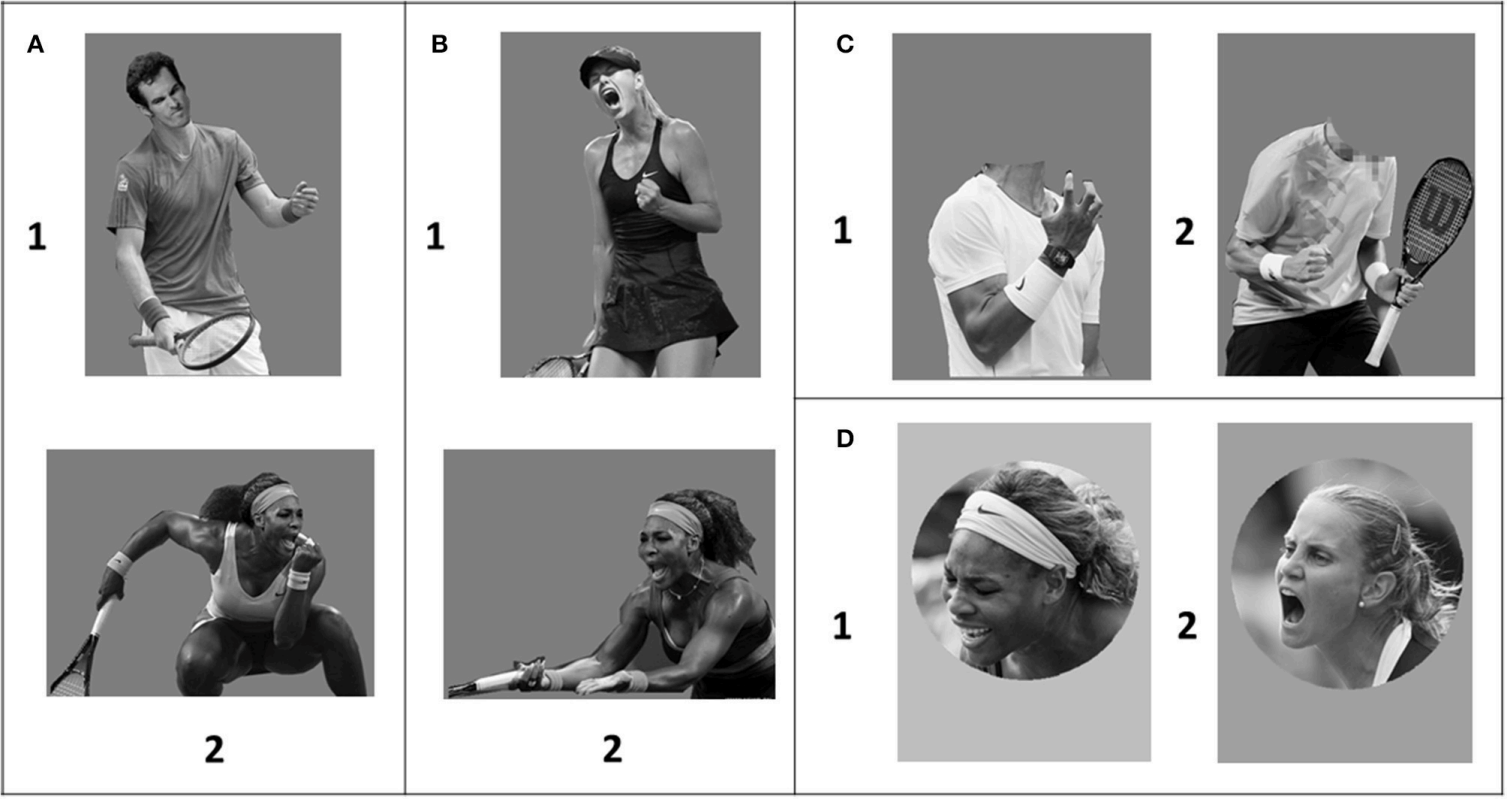
**(A)** Examples of congruent pictures: (1) losing-face-losing-body; (2) winning-face-winning-body. **(B)** Examples of incongruent pictures: (1) winning-face-losing-body; (2) losing-face-winning-body. **(C)** Examples of pictures: (1) isolated winning body; (2) isolated losing body. **(D)** Examples of pictures: (1) isolated losing face; (2) isolated winning face.

Images of tennis athletes, depicting the transient peak-intense reactions to winning or losing a point in high-stake competitions, were selected from Google (Same key words were used as Aviezer et al., 2012b). Every image was digitally manipulated using photo-editing software to create four image categories:

1. Isolated-face: nine images in total, five losing faces (three females and two males) and four winning faces (two females and two males) (see Figure 1D);
2. Isolated-body: eight images in total, four winning bodies (two females and two males) and four losing bodies (two females and two males) (see Figure 1C);
3. Face-body congruent images: three images in total (two losing and one winning) (see Figure 1A);
4. Face-body incongruent images: seven images in total (four losing-face-winning-body and three winning-face-losing body) (see Figure 1B).

All the participants were unaware of the manipulation. Pictures were presented in grayscale, with a gray background. The size of the vertical stimuli was 350 × 533 pixels and the size of horizontal stimuli was 533 × 350 pixels.

#### Procedure

The participants were directed into the laboratory. After signing the informed consent forms, they were seated in front of an eye-tracking device positioned 64 cm in front of them, with their head placed on a chin-rest. A nine-point calibration was then performed, during which the participants were required to follow the calibration point as it moved over the screen to ensure that eye gaze data were adjusted for movement. Calibration was repeated before each block. Eye movements were recorded with Tobii T120, at the sample rate of 120 Hz. All of the instructions for the study were given by computer.

The study comprised three blocks: isolated face block, isolated block, and face-body compound block (with both face-body congruent and incongruent images). All the images were randomly presented in each block. The order of three blocks was counterbalanced across all participants. The participants were given the instruction to look carefully at the images and evaluate the emotional states of the athletes in the images after they disappeared. Each image was presented for 5000 ms followed by an evaluative scale. Since all the pictures showed high arousal, participants were only asked to evaluate the valence: this refers to the pleasant or unpleasant state, with 1 for extremely unpleasant, 5 for neutral, and 9 for extremely pleasant.

### Results

The participants rated isolated facial and bodily expressions and face-body compounds; their fixation patterns (fixation duration and fixation count) were recorded simultaneously. The original ratings of valence varied from 1 to 9. We transferred them to −4 to 4 by subtracting 5 from the original ratings. Thus, the ratings below 0 stood for negative, those above 0 stood for positive, and 0 represented neutral.

#### Accuracy

If the response (positive/negative/neutral) was consistent with the emotion shown in the image, it was recorded as correct; if not, it was recorded as incorrect. Since the face-body incongruent images displayed two different emotions simultaneously, their accuracy cannot be calculated. We will, therefore, present the results for incongruent images separately.

Except for the face-body incongruent images, the overall accuracy was 81%, which was significantly above chance performance: in one-sample *t*-test, *t*_(31)_=13.88, *p* < 0.01. Breaking down accuracy for the three kinds of images: isolated face was 66%, isolated body was 89%, and face-body congruent was 88%. A paired sample *t*-test was conducted. The accuracy for isolated face was significantly lower than the isolated body images, *t*_(31)_ = −7.79, *p* < 0.01; and the face-body congruent images, *t*_(31)_ = −5.74, *p* < 0.01; but significantly above chance, *t*_(31)_ = 6.28, *p* < 0.01. There were 192 face-body incongruent trials in total, of which only 18 trials were evaluated corresponding to face, representing 9.4% of the total.

However, it should be claimed that the overall accuracy for isolated face images (66%) is driven by the high accuracy for the losing face. We calculated the accuracy for the winning face and losing face separately: the results showed that the mean accuracy for the losing body is 92%, which is significantly higher than chance level (50%), *t*_(31)_ = 20.00, *p* < 0.01; but the mean accuracy for the winning body is 39%, which is marginally significantly below chance level, *t*_(31)_ = −1.88, *p* = 0.07. Thus, the 66% accuracy of peak facial expression may not indicate the diagnosability. Instead, it indicated that participants tended to take both winning and losing peak facial expressions as lose.

#### Emotional ratings

##### Isolated face and isolated body image

**Figure 2:**
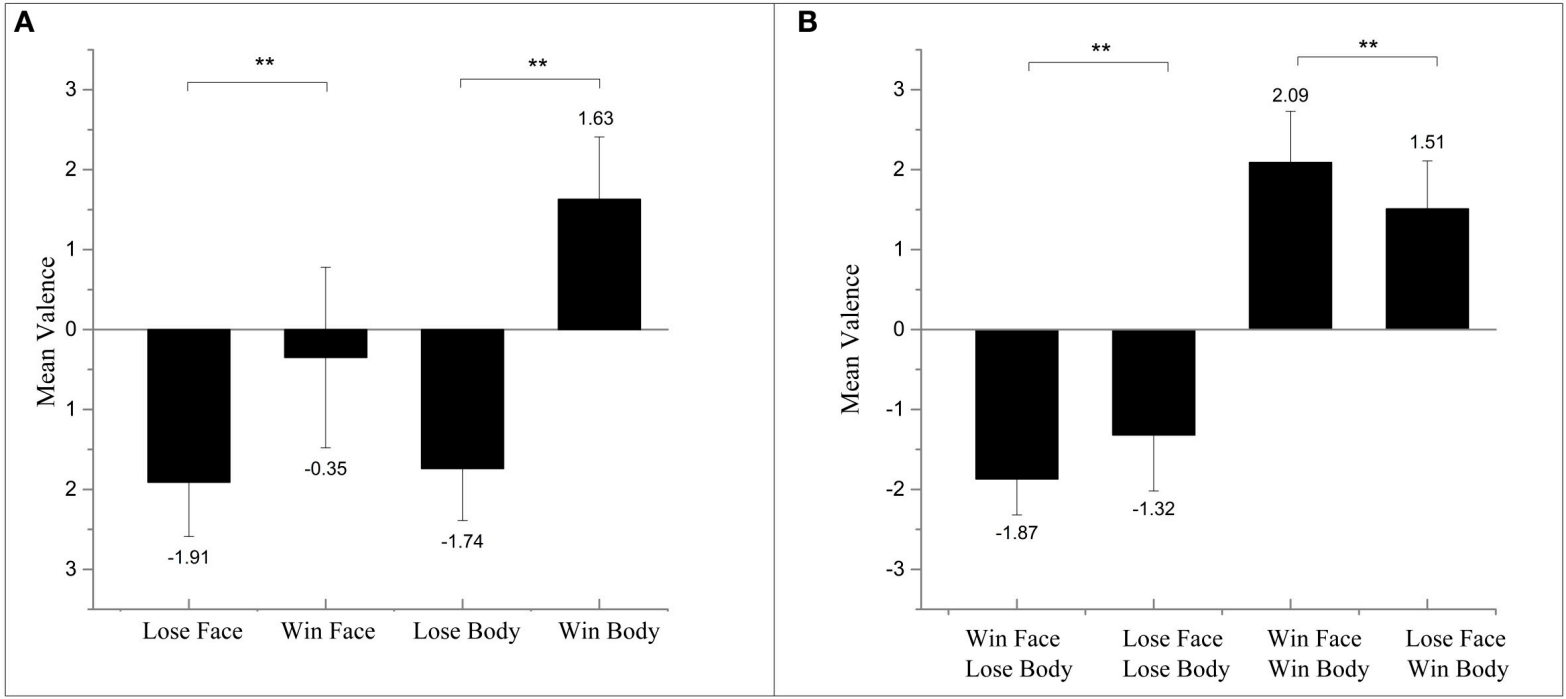
**(A)** Results of mean valence ratings for images of isolated face and isolated body. **(B)** Results of mean valence ratings for images of face and body compounds. **p < 0.01.

Participants were able to correctly evaluate the valence of isolated body images: they succeeded in rating winning bodies as positive and losing bodies as negative. However, they failed to judge the emotional valence when faces were shown alone. Specifically, the participants evaluated both losing faces and winning faces as negative when measuring the emotional ratings (see Figure 2A).

##### Face-and-body images

A 2 (Body: losing/winning) × 2 (Face: losing/winning) repeated-measure ANOVA on valence ratings revealed a main effect of the body, *F*_(1, 31)_ = 114.21, *p* < 0.01, $\eta_{\text{p}}^{2}=$ 0.80, suggesting that judgments of peak emotions were mostly accordant to bodies regardless of the congruency. The recognized affective valence of face-body compounds shifted mainly depending on the body. Furthermore, in accordance with the previous study of Aviezer et al. (2012b), the interaction between the two factors reached significance: *F*_(1, 31)_ = 20.53, *p* < 0.01, $\eta_{p}^{2}=$ 0.39. The subsequent paired t-test showed that images with winning faces were rated as more extreme. Congruent winning images were rated as significantly more positive than losing face-winning body, *t*_(31)_ = 3.23, *p* < 0.01, and winning-face-losing-body images were rated as significantly more negative than congruent losing: *t*_(31)_ = −3.37, *p* < 0.01 (see Figure 2B).

#### Eye movement

Eye movements were recorded from 23 participants. To better reveal the observation processes, we removed the blocks whose recording samples were below 70%. Samples are the index of the quality of recording as a percentage, which is calculated by correctly recognized numbers of eye movement samples. One hundred percent means both eyes were found throughout the recording; 50% means only one eye was fully recorded or both eyes during half duration. Since we were interested in the relative contributions of body and face to the emotion recognition, analysis on body-and-face images was conducted in terms of the number of fixations and fixation duration. There remained 17 blocks for face-body compound.

**Figure 3:**
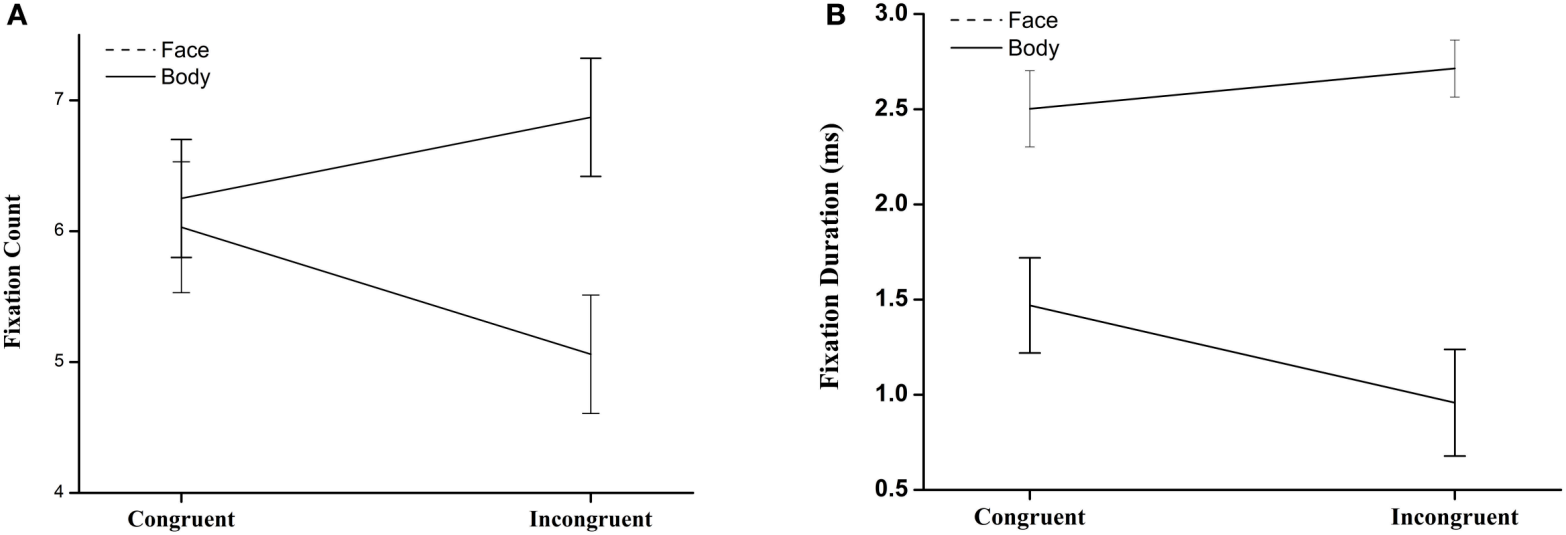
**(A)** Number of fixation for Regions of Interest (ROI) of face and body in face-body compounds. **(B)** Fixation duration fixation for Regions of Interest (ROI) of face and body in face-body compounds.

We defined two regions of interest (ROI): the face and the body in body-and-face compound images. The average number of fixations per ROI for each image type was calculated. A 2 (ROI: Body/Face) × 2 (Congruency: Congruent/Incongruent) repeated-measure ANOVA was conducted. There were no significant main effects of ROI or congruency. The interaction between ROI and Congruency was significant: *F*_(1, 16)_ = 9.87, *p* < 0.01, $\eta_{p}^{2}=$ 0.38. A further paired *t*-test revealed that there was no significant difference between body and face in congruent situations, *t*_(16)_ = −0.33, *p* > 0.05; while the number of fixations on face was significantly higher than on body in incongruent images, *t*_(16)_ = −2.39, *p* < 0.05 (see Figure 3A). The same analysis was conducted on the fixation duration, revealing a significant main effect of ROI, *F*_(1, 16)_ = 44.38, *p* < 0.01,$\eta_{p}^{2}=$ 0.74, and congruency, *F*_(1, 16)_ = 8.01, *p* < 0.05, $\eta_{p}^{2}=$ 0.33, and significant interaction between them, *F*_(1, 16)_ = 29.25, *p* < 0.01, $\eta_{p}^{2}=$ 0.65. A further paired *t*-test revealed significant differences between body and face in both congruent, *t*_(16)_ = −0.49, *p* < 0.01, and incongruent situations, *t*_(16)_ = −2.39, *p* < 0.05 (see Figure 3B). The results of both the fixation count and the fixation duration suggested that the participants displayed distinct gaze patterns for congruent and incongruent images.

The emotional ratings of the 17 remaining participants included in the analysis of eye-tracking were also analyzed. They produced similar results: a significant main effect of the body, *F*_(1, 16)_ = 98.08, *p* < 0.01, $\eta_{p}^{2}=$ 0.87. The interaction between the two factors was also significant, *F*_(1, 16)_ = 19.92, *p* < 0.01, $\eta_{p}^{2}=$ 0.57. A subsequent paired *t*-test showed that congruent winning images were rated as significantly more positive than losing face-winning body images, *t*_(16)_ = 3.48, *p* < 0.01, and winning face-losing body images were rated as significantly more negative than congruent losing, *t*_(16)_ = −2.57, *p* < 0.01.

Moreover, to solve the possible problem caused by an unequal number of materials, we randomly selected three images from each group (isolated-face, isolated-body, face-body congruent, and face-body incongruent) to repeat the same analysis on fixation duration and number of fixations. The results were almost the same. For fixation duration, the main effects of ROI [*F*_(1, 16)_ = 35.73, *p* < 0.01, $\eta_{p}^{2}=$ 0.69], congruence [*F*_(1, 16)_ = 11.29, *p* < 0.01, $\eta_{p}^{2}=$ 0.41], and the interaction effect between ROI and congruence were all significant [*F*_(1, 16)_ = 10.22, *p* < 0.01, $\eta_{p}^{2}=$ 0.39]. A further paired *t*-test revealed a significant difference between body and face in both congruent*, t*_(16)_ = −6.29, *p* < 0.01, and incongruent situations*, t*_(16)_ = −4.90, *p* < 0.01. For fixation duration, neither the main effects of ROI nor those of congruence were significant, but the interaction between these two effects showed the trend to reach significance, *F*_(1, 16)_ = 3.23, *p* = 0.09, $\eta_{p}^{2}$ = 0.17.

### Discussion

Experiment 1 aimed to investigate the explicit recognition of peak facial and bodily expressions and the relative contribution of body and face during the emotion recognition process. The emotion rating results were consistent with the principal previous study (Aviezer et al., 2012b), revealing that faces were not able to provide sufficient valence information in peak emotion situations.

One of the most interesting findings in Experiment 1 was that the participants showed different gaze patterns to face-body congruent and incongruent images. This was reflected by the significant interaction between ROI and congruency, with larger distinctions between ROIs in incongruent images than in congruent images, in terms of both fixation duration and number of fixations.

We query why the distinct gaze patterns appeared. It could be assumed that if the participants were unable to discriminate valence (both explicitly and implicitly) from intense facial expressions, there would not be “congruent” or “incongruent” to them. Since ambiguity of peak facial expressions could not provide any valid emotional information to match or mismatch with the bodily gestures. But in fact, participants did display distinct eye-gaze patterns to the congruent and incongruent groups. One possible explanation of the different gaze patterns was that people could perceive specific emotional information from the intense facial expressions, maybe in an unconscious way. To further investigate the unconscious perception process of facial expressions, Experiment 2A and 2B were conducted.

## Experiment 2A

According to Murphy and Zajonc (1993) Affective Primacy Theory, the emotional reaction to a stimuli could be activated with minimal stimuli input and few cognitive resources. Consistent with this, previous studies have shown that people can process faces of different valence in the absence of consciousness. Neurons in the superior colliculus are capable of responding to rapid visual input and producing distinct responses to facial expressions without any conscious experience (Blair, 2003). In essence, emotion perception is highly automatic, outside consciousness, and prior to other cognition and perception (Massar and Buunk, 2009). In Experiment 2A, we tested the implicit emotion perception process of peak emotion facial expressions.

### Materials and methods

#### Participants

Twenty-eight undergraduates (nine males and 19 females; *M* = 22.08 years, *SD* = 1.20, range: 19–24 years) gave informed consent and received small gifts for their participation. All were right-handed and reported normal or corrected-to normal vision.

#### Materials

The methods employed in Experiment 1 were used to search for and collect more images from Google. Twenty losing bodies and 20 winning bodies were used as targets, each presented three times; 30 losing faces and 30 winning faces were used as priming stimuli, each appearing twice; and ten athletes' images with a mosaic filter were used as masks, each displayed 12 times. All the faces and bodies were moved from the original images to a gray background.

#### Procedure

The participants seated in front of a computer. After signing the informed consent forms, they were asked to undertake a subliminal affective priming procedure adapted from Li and Lu (2014). Throughout the experiment, all instructions were given on the screen. The study consisted four blocks, with the first block used as a practice block and the other three as experimental blocks, each containing 30 trails. The four blocks (two congruent blocks and two incongruent blocks) were counterbalanced, so that the practice block could be either a congruent block or an incongruent block.

**Figure 4:**
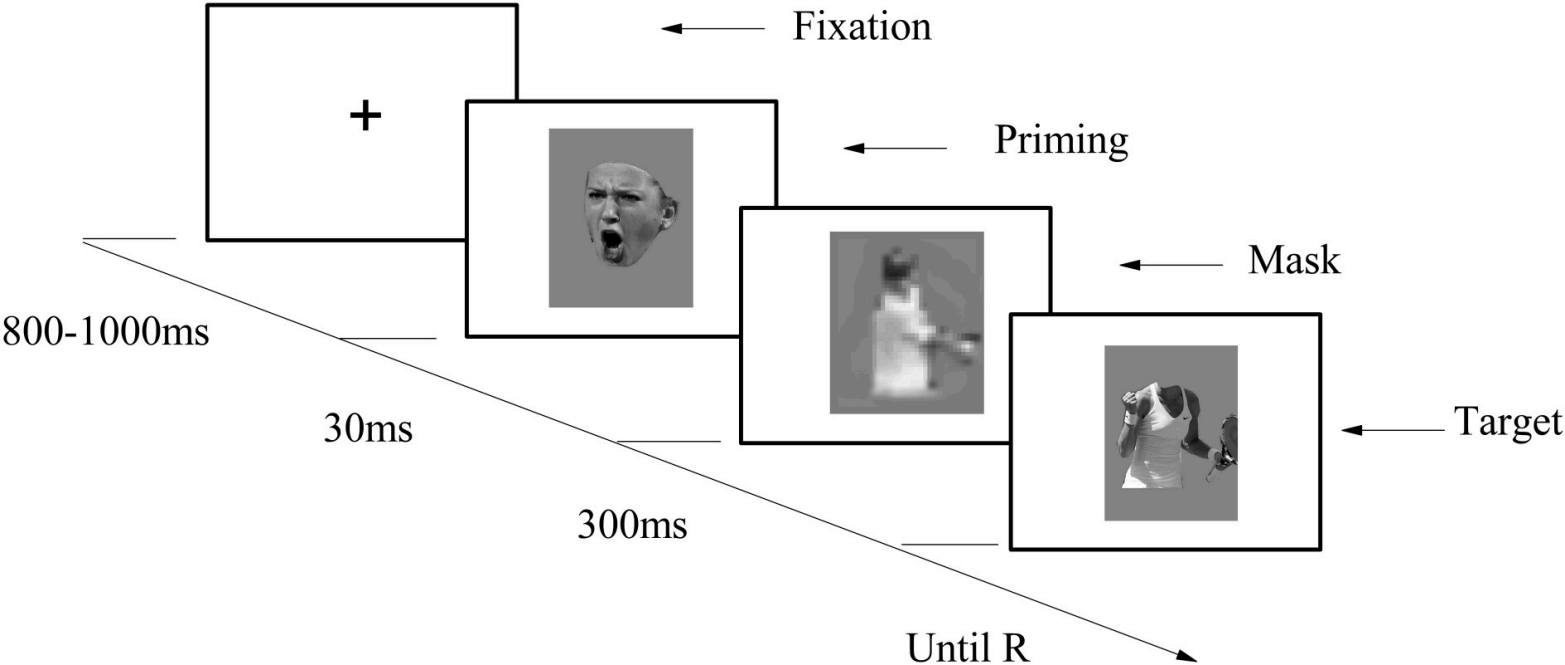
**Procedure of Experiment 2A**.

The sequence of events in a trial is depicted in Figure 4. Each trial started with a “+” randomly presented for 800–1000 ms at the center of the screen; then a priming stimuli was presented for 30 ms as a flash; a mask was then presented for 300 ms; finally, the target stimuli appeared on the screen, remaining visible until the participant responded. The participants were told to rate the valence of emotional body gestures by pressing numbers 1–9 on the keyboard, with 1 standing for extremely unhappy, 5 for neutral, and 9 for extremely happy. They were also informed that the masks were used to prevent the interruption between two sequentially presented trials, for which reason they did not have to pay attention to them. Immediately after completion of the subliminal affective priming task, participants were asked about the priming stimulus, namely: “*Have you noticed anything strange or curious during the experiment?”* (Montoro et al., 2014) No one reported having seen the priming stimulus.

### Results

All the practice blocks were removed from the data analysis; thereby, 14 congruent blocks and 14 incongruent blocks were removed. No other data were removed.

#### Reaction time (RT)

**Figure 5:**
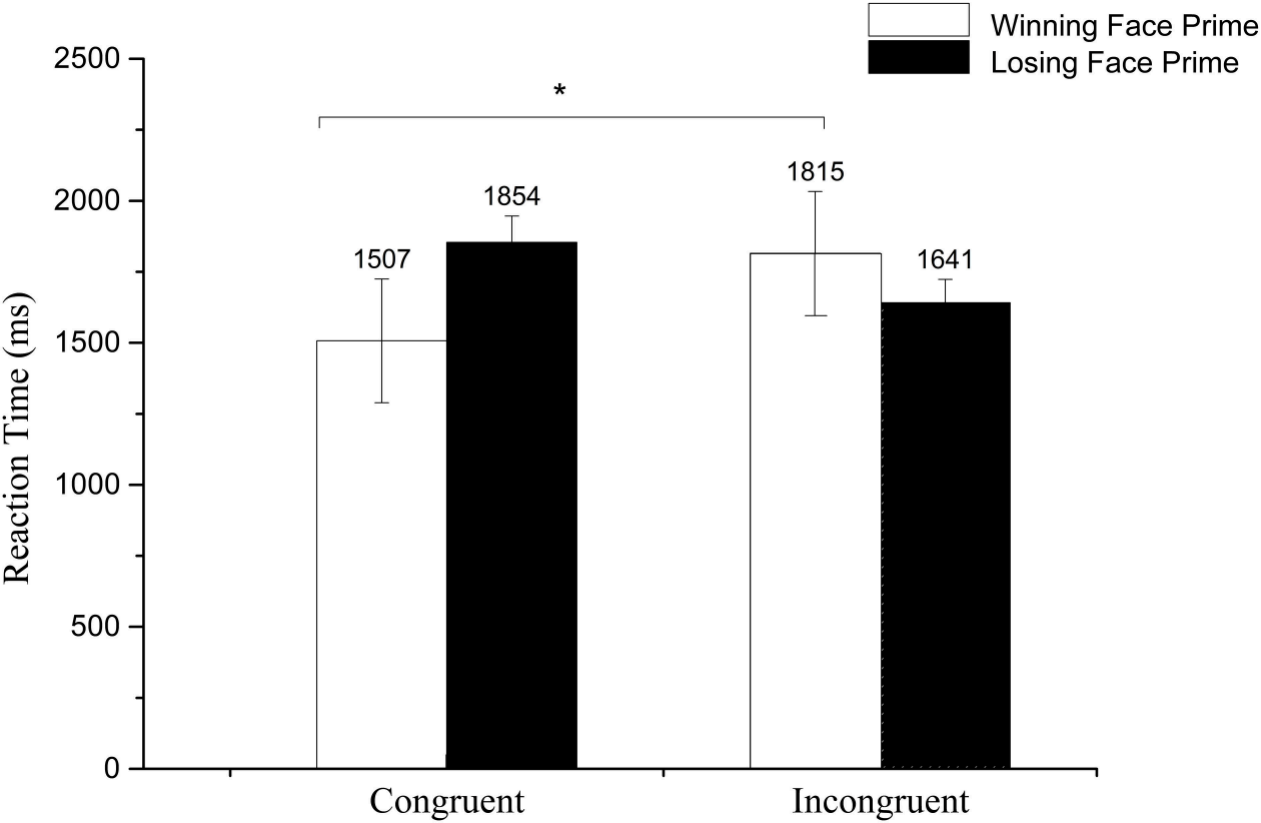
**Mean reaction time to losing and winning face primes in congruent and incongruent conditions**. *p < 0.1.

We calculated the average reaction time to the target and conducted a 2 (Prime valence: losing/winning) × 2 (Congruence: congruent/incongruent) repeated ANOVA. A significant main effect of prime valence was found, *F*_(1, 27)_ = 4.67, *p* < 0.05, $\eta_{p}^{2}=$ 0.15, with a longer reaction time to losing face primes than to winning face primes. The interaction between prime and congruence reached significance, *F*_(1, 27)_ = 13.89, *p* < 0.01,$\eta_{p}^{2}=$ 0.34. Further paired *t*-test revealed a longer RT for winning faces under the incongruent context than the congruent context, *t*_(27)_ = 2.23, *p* < 0.05, but no significant difference for the losing faces, *t*_(27)_ = 0.712, *p*>0.05 (see Figure 5).

#### Emotional ratings

We calculated the average emotional ratings to the target and conducted a 2 (Prime valence: losing/winning) × 2 (Target valence: losing/winning) repeated ANOVA. Neither significant main effects nor the interaction between two factors were obtained.

### Discussion

Experiment 2A investigated the subliminal priming effect of peak facial expressions. An affective priming effect occurred when the emotional information of the prime and the probe were the same, measured by the shorter reaction time (Murphy and Zajonc, 1993). The significant RT interaction between prime and target revealed that the subliminal affective priming effect occurred in the winning body context. The reason for us not finding the subliminal affective priming effect of losing body in this study might be that we did not limit the time to react to the targets; consequently, it took more time to react to losing bodies, which possibly diminished the subtle differences between the two conditions.

One limitation of our Experiment 2A is that no time limitation was set to prevent the long-time interruption to the subliminal affective priming effect, although the participants were informed to react as quickly as possible. Longer exposure to targets may diminish the priming effect on reaction. In order to solve the problem of experiment 2A, we conducted Experiment 2B to further examine the unconscious perception of peak facial expressions.

## Experiment 2B

In order to prove the validity of the subliminal affective priming effect of peak expressions, we adopted a revised paradigm and an additional strict awareness test to make sure that the subliminal affective priming effect found in Experiment 2A was truly caused by the invisible primes and the influence of peak facial expression primes on the reaction to targets occurred through an unconscious process.

### Materials and methods

#### Participants

Forty-three undergraduates (20 males and 23 females; *M* = 22.70, *SD* = 1.50, range: 21–24 years) gave informed consent and received small gifts for their participation. All were right handed and reported normal or corrected-to normal vision.

#### Materials

In the main experiment, all the stimuli were the same as Experiment 2A, with exception that the mask stimuli were changed to a noise image.

**Figure 6:**
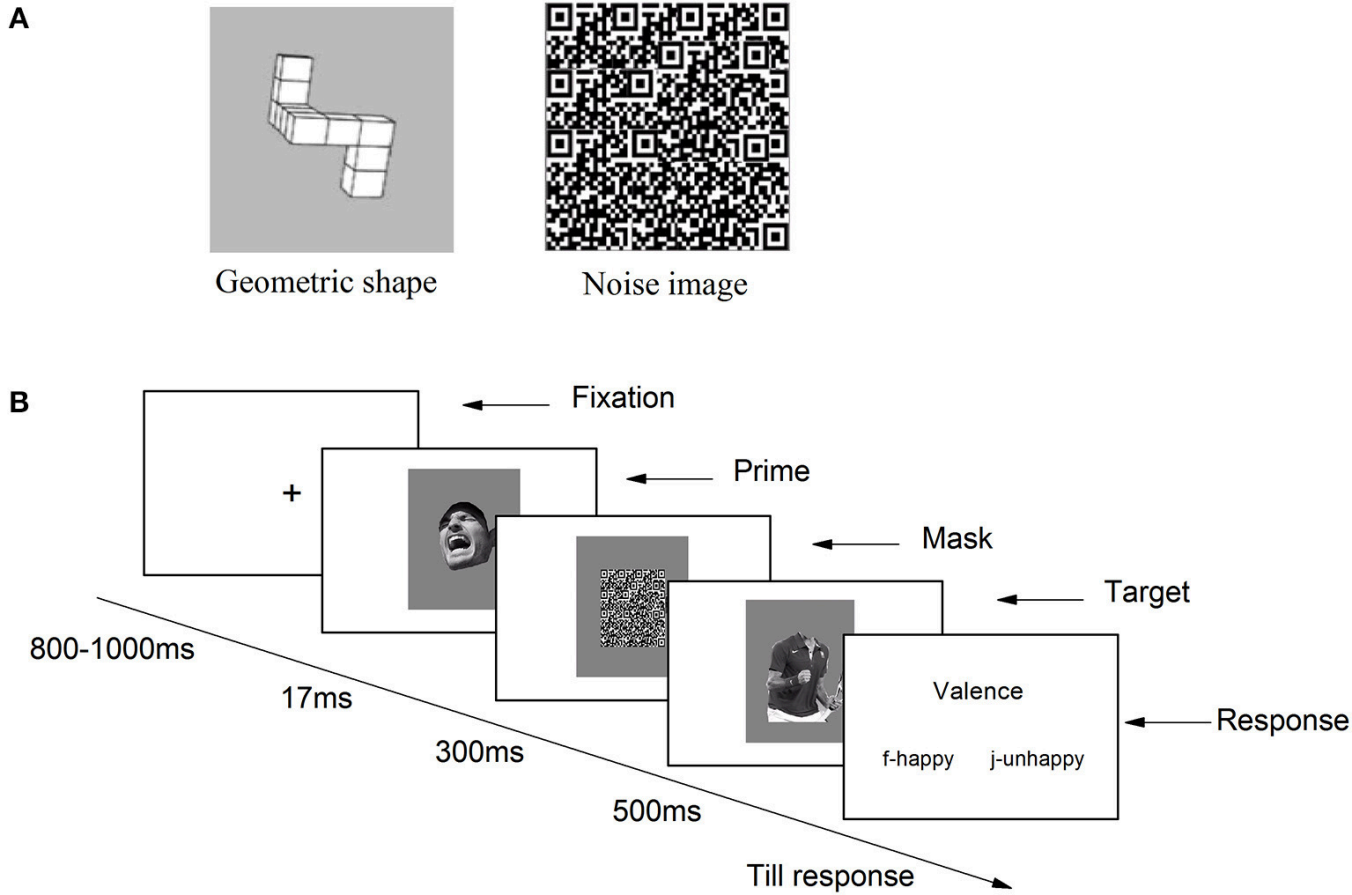
**(A)** Examples of noise image and image of geometric shape used; **(B)** Revised procedure of subliminal affective priming task used in Experiment 2B.

In the awareness test, there were 120 trials. Sixty trials were face primes (30 winning faces and 30 losing faces, the same as Experiment 2A) and the other 60 trials were primed by an image of geometric shape (see Figure 6A).

#### Procedure

The same paradigm as Experiment 2A was used in Experiment 2B, and we modified the experimental procedure in the following ways to make sure that the prime stimuli were truly unseen. First, the primes were presented for 17 ms instead of 30 ms. Second, the mask stimuli were changed from athletes' images with a mosaic filter to the noise images to provide a better masking effect. Third, the targets in current experiment were presented for 500 ms instead of until response and responses were made after the targets offset. Forth, in Experiment 2A, we inferred that the potential priming effect of losing face was hindered by the long reaction time. Thus, the task in present experiment was made easier by asking participants to discriminate the valence of target by pressing “f” or “j” on keyboard, with “f” for happy, “j” for unhappy (see Figure 6B).

In addition, we added a prime discrimination task as awareness test to measure the extent to which participants were aware of the prime pictures. The sequence was exactly the same as main experiment, except that participants were told to discriminate whether the primes were faces or non-face objects. Participants pressed “f” if they thought the prime was a face, and the “j” if they thought the prime was a non-face object. The non-face objects were consisted of geometrical shapes.

### Results

#### Prime awareness test

Following the studies of Almeida et al. (2013), we calculated the accuracy on the prime awareness task to select participants in present experiment. The participants whose performance met the following criteria were included in the main analysis: (1) they did not report to notice anything strange or curious during the experiment; (2) their overall accuracy and d' to the prime are no different from 50% and 0 (*z*-test for one proportion); (3) the accuracy on face prime and non-face prime is not significantly different from chance level (*z*-test for one proportion); (4) the accuracy between the face prime and non-face prime trials do not significantly differ from each other; (5) the accuracy for winning face primes and losing face primes do not differ from each other significantly.

**Figure 7:**
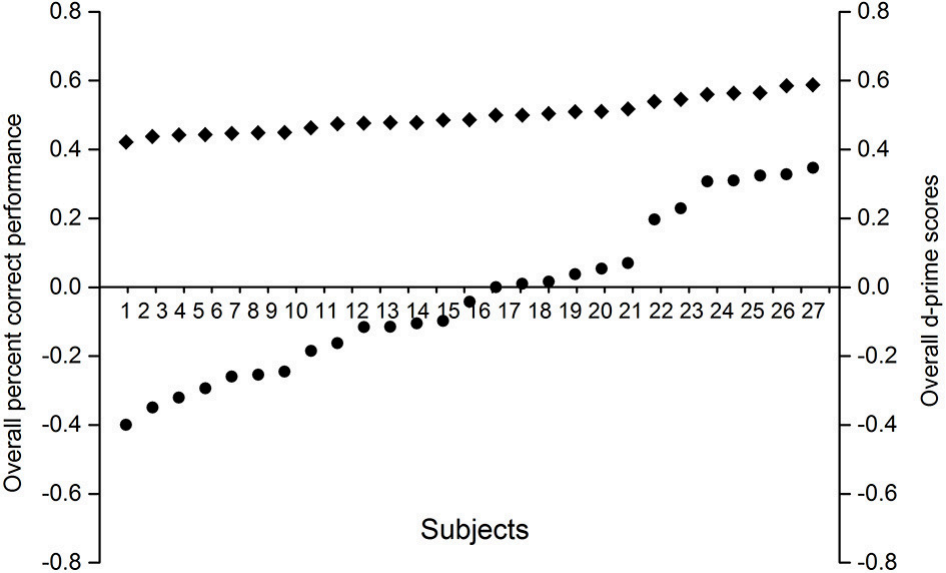
**Individual overall proportions correct and d' scores on the prime awareness measures**. Filled circles correspond to the individual d' scores, whereas filled diamonds correspond to the individual percent correct scores.

According to the criteria above, a total of 16 participants were excluded from the main analysis, and there were 27 participants entered the main analysis. The percent correct performance for the participants did not differ from 50% (see Figure 7), which indicated that they did not experience any conscious perception of prime stimuli.

#### Reaction time

**Figure 8:**
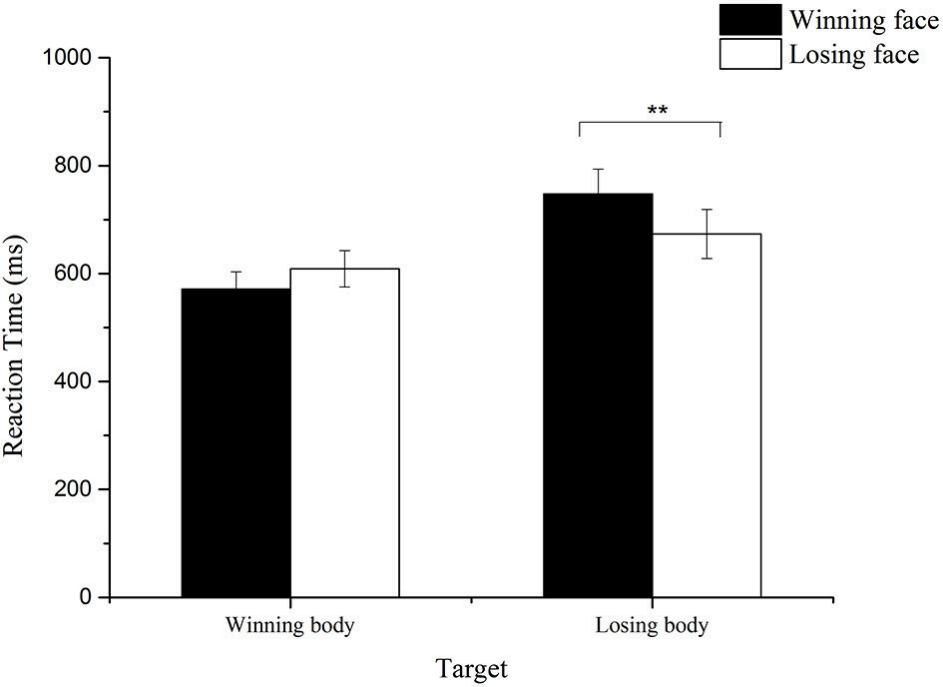
**Mean reaction time to the winning and losing body targets with congruent and incongruent primes**. **p < 0.01.

For the remaining 27 participants, false response trials and the trials whose reaction time was below 100 ms or exceeded three standard deviations were removed (6% of the total number of trials). We calculated the average reaction time for each condition and conducted a 2 (Prime: wining/ losing) × 2 (Target: wining/losing) repeated measure ANOVA. A significant main effect of target was obtained, *F*_(1, 26)_ = 12.10, *p* < 0.01, $\eta_{p}^{2}=$ 0.32, with longer reaction time to losing body target than winning body target. The interaction between the prime and target also reached significance, *F*_(1, 26)_ = 10.35, *p* < 0.01, $\eta_{p}^{2}=$ 0.29. Further paired *t*-test showed that for losing body target, participants showed significantly longer reaction time when primed by winning faces than when primed by losing faces, *t*_(26)_ = 2.59, *p* < 0.01; for winning body target, the reaction time increased when primed by losing faces, compared with when primed by winning faces, which is marginally significant, *t* = 1.93, *p* = 0.06 (see Figure 8).

### Discussion

In the present study, we used strict criteria to ensure that the participants did not experience any conscious perception of the subliminally presented primes. Moreover, in order to examine the priming effect of losing faces, which was not found in Experiment 2A due to the long reaction time, we shortened the exposure time of target and simplified the experimental task by asking participants to distinguish the valence of target with only two keys on the keyboard. Results showed that the peak expressions could influence the reaction time to the body targets, with faster reaction to congruent trials and slower reaction to incongruent trials, which indicated that the peak facial expressions could be unconsciously perceived.

## General discussion and conclusion

In the three experiments of the current study, we attempted to extend the previous research on peak facial expression perception and recognition. More specifically, we investigated peak facial expression recognition at both explicit and implicit levels. In Experiment 1, we aimed to explore whether peak facial expression could be explicitly recognized. To address this issue, we presented the participants with images of peak emotion and asked them to make emotional valence evaluations. In addition, we recorded eye movement during the observations, to investigate the contributions of face and body to peak emotion recognition. The valence rating results of Experiment 1 showed that isolated peak facial expressions were not diagnostic at a conscious level. Indeed, the participants tended to perceive both winning and losing expressions as losing. Conversely, the emotional valence of the body could be easily recognized and largely influenced the valence judgment of face-body compounds. More specifically, the valence judgment of face-body compounds shifted in accordance with the body's affective valence. The eye tracking results revealed that participants exhibited more fixation toward face when confronted with conflicting emotional information. In Experiment 2A and 2B, the subliminal affective priming tasks were adopted to examine the implicit perception of peak facial expressions. We used isolated body as the target and isolated face as the prime. All the images were of peak emotions. A subliminal affective priming effect was found to some degree, given the evidence that participants responded faster to winning body when primed with winning face, and slower to winning body when primed with losing face.

Eye movement is always regarded and used as an objective measure of attention (Kim and Lee, 2016), and the attention oriented to particular parts of faces is thought to affect emotion recognition. The different gaze patterns—when and where the participants looked—indicated the information entering the visual system and the strategies adopted in emotion perception (Watanabe et al., 2011). It has been assumed that increasing fixation toward specific regions could improve emotion recognition accuracy, despite the notions that scanning patterns of faces is not the only factor to determine the accuracy (van Asselen et al., 2012). In the study of Adolphs et al. (2005), they found that the deficit in fear recognition displayed by a patient with bilateral amygdala damage was due to the lack of spontaneous fixation toward the eye-region. When the patient was instructed to look at the eyes, their fear recognition returned to normal. Watanabe et al. (2011) suggested that females are more sensitive to emotions because they tend to focus more on the main parts of the face (eyes, nose, and mouth).

The significant main effect of the region of interest showed that faces attracted more attention than bodies, which highlighted the specificity of face. More fixation on faces indicated people's intrinsic tendency to look at the face for emotion information. Rosenthal et al. (1977) found that face plays a dominant role in emotion recognition by demonstrating that the channels of vocal tone, body, and face contribute to emotion recognition in a ratio of 1:2:4. Besides the function of conveying emotion information, there are other factors that lead to more fixation on the face. First, the physical properties of the face are so complex that it requires more cognitive resources to interpret. Secondly, the human face is an enormously important source of information regarding, for example, age, sex, race, and intention. Although the participants were not instructed to identify these characteristics, these processes occur automatically. Moreover, humans exhibit an innate bias for face perception, even in early childhood (Valenza et al., 1996).

The eye movement results in Experiment 1 revealed that incongruent images resulted in more fixation to the face and less fixation to the body compared with congruent images, thus concurring with the results of Shields et al. (2012), whose task was the same as ours. In their study, they adopted photographic images of moderate basic emotions (happy, afraid, angry, and sad) to create face-body incongruent compounds, and asked participants to choose which emotion the person in the image was displaying, while simultaneously recording their eye movement. Though the participants were inclined to exhibit more attention to faces in both cases (for moderate emotions and peak emotion)—in essence, more attention was focused on faces when confronted with conflicting information—the emotion recognition results they recorded are quite different from those of our study. The participants who viewed moderate emotions were more likely to choose emotions shown in the face (for some materials) or equally likely to choose emotions shown in the body and face (for the others). Conversely, for peak emotions in our study, the body gestures were more frequently used as the emotional cues for valence judgment.

We propose the following explanation to account for why, in contrast to moderate emotions, body expression biases emotion recognition toward the emotion conveyed by body, despite more fixation on the face in peak emotion recognition. It may be reasoned that bodily gestures are equally crucial as facial expressions for survival, as they serve an adaptive function to cope with the current situation. Many studies have demonstrated that emotional valence tends to connect with the approach-withdraw motivations (Cacioppo et al., 1999; Harmon-Jones, 2003a,b; Carver, 2004; Harmon-Jones et al., 2013). Indeed, the dimension of approach-withdraw is always presented by body gestures. Approach is inferred from stretching, opening, and moving forward, which indicates movement toward others (winning gestures in our study). Conversely, withdraw is inferred from bowing, closing, and moving downward or backward, indicating the tendency of moving away from the situation (the losing gesture in our study; James, 1932; De Meijer, 1989). Face is not the only factor or determiner for emotion perception. Indeed, people attempt to incorporate information from multiple channels (Bogart et al., 2014). It is still controversial which modality is dominant when presenting diverse modalities, especially when the information they convey is equally clear. However, in the case of peak emotion, information obtained from bodily gestures is strong and clear, while information from facial expressions is mostly vague. As Van den Stock et al. (2007) indicated, the influence of body expression increases when the facial ambiguity is high, and decreases when the facial ambiguity reduces: this may explain why body gestures play a more important role in peak emotion situations.

Many studies have been conducted regarding the implicit perception of moderate basic emotions; however, to date, research has rarely investigated the implicit processes of peak facial expressions. Most prior studies have demonstrated that moderate emotions can be perceived unconsciously. By using event-related potential (ERP) technology, researchers have supported the early perception of emotional information, indexed by components of P1 and N170, occurring at mean latency of 90 and 170 ms (Eimer and Holmes, 2002; Batty and Taylor, 2003; Eimer et al., 2003; Aguado et al., 2012; Hinojosa et al., 2015). In the study of Aguado et al. (2012), they reported an increased amplitude of N170 at temporal sites in the presence of angry facial expressions.

In the current study, we conducted a subliminal affective priming task, with peak facial expressions as primes and body gestures as targets to investigate the unconscious perception of peak facial expressions. Implicit perception of peak facial expressions can be assessed by analyzing the facilitated and inhibited reaction to emotional targets (Dimberg et al., 2000; De Gelder et al., 2002). The participants responded faster to winning bodies when primed with winning faces than when primed with losing faces. Also, participants responded slower to losing bodies when primed with winning faces than when primed with losing faces. The results of Experiment 2A and 2B revealed that isolated peak facial expressions could be perceived unconsciously. Our studies sought to contribute to the literature on the emotion recognition of peak facial expressions. Our work provides a foundation for several new considerations in peak facial expression recognition. Further studies could consider the use of dynamic expressions. In the case of emotion recognition, especially peak emotion recognition, perceiving dynamics is of great importance, since observers perceive emotion dynamically, and dynamic expressions are thought to be easier to recognize than static expressions (Atkinson et al., 2004). When viewing two successive presentations of facial expressions with implied motion, participants might fail to recognize the difference between them (Freyd, 1983). However, the motion becomes more salient for peak emotion, since it is always transiently presented and the face resumes diagnosability shortly after the peak intensity subsides. Further studies should address the possibility that peak facial expressions might be more informative if perceived dynamically over the second or two in which they appear and disappear. Moreover, further studies could also investigate whether there are any possibilities to recognize peak emotions explicitly by using oxytocin, since one previous study (Perry et al., 2013) showed that neuropeptide oxytocin was able to improve sensibility to detect the subtle differences between similar facial expressions.

## Author contributions

Concept and design of study: YW, RX, XL, LL. Data acquisition, analysis, and interpretation: YW, RX. Drafting the work or revising it critically for important intellectual content: YW, RX. Final approval of the version to be published: YW, RX, XL, LL. Agreement to be accountable for all aspects of the work in ensuring that questions related to the accuracy or integrity of any part of the work are appropriately investigated and resolved: YW.

### Conflict of interest statement

The authors declare that the research was conducted in the absence of any commercial or financial relationships that could be construed as a potential conflict of interest.
